# Prevalence of chimerism after non-myeloablative hematopoietic stem cell transplantation

**DOI:** 10.1590/s1516-31802009000500002

**Published:** 2010-02-03

**Authors:** Azulamara da Silva Ruiz, Maria de Lourdes Ferrari Chauffaille, Solivanda Trindade Alves, José Salvador Rodrigues de Oliveira

**Affiliations:** I BSc. Postgraduate student, Division of Hematology and Transfusion Medicine, Universidade Federal de São Paulo - Escola Paulista de Medicina (Unifesp-EPM), São Paulo, Brazil.; II MD, PhD. Associate professor, Department of Medicine. Division of Hematology and Transfusion Medicine, Universidade Federal de São Paulo - Escola Paulista de Medicina (Unifesp-EPM), São Paulo, Brazil.; III MD. Physician in the Division of Hematology and Transfusion Medicine, Hospital Santa Marcelina, São Paulo, Brazil.; IV MD, PhD. Associate professor, Department of Medicine, Division of Hematology and Transfusion Medicine, Universidade Federal de São Paulo - Escola Paulista de Medicina (Unifesp-EPM); and coordinator of the Division of Hematology and Transfusion Medicine, Hospital Santa Marcelina, São Paulo, Brazil.

**Keywords:** Variable number of tandem repeats, Hematologic neoplasms, Bone marrow transplantation, In situ hybridization, fluorescence, Chimerism, Leukemia, Repetições mini-satélites, Neoplasia hematológicas, Transplante de medula óssea, Hibridização in situ fluorescente, Quimerismo, Leucemia

## Abstract

**CONTEXT AND OBJECTIVE:**

Non-myeloablative hematopoietic stem cell transplantation (NMA-HSCT) is performed in onco-hematological patients who cannot tolerate ablative conditioning because of older age or comorbidities. This approach does not completely eliminate host cells and initially results in mixed chimerism. Long-term persistence of mixed chimerism results in graft rejection and relapse. Involvement of graft-versus-host disease is concomitant with complete chimerism and graft-versus-tumor effect. The aim of this study was to evaluate the prevalence of chimerism in onco-hematological patients who underwent NMA-HSCT.

**DESIGN AND SETTING:**

Observational clinical study on chimerism status after human leukocyte antigen-identical NMA-HSCT at the Discipline of Hematology and Hemotherapy of Universidade Federal de São Paulo.

**METHODS:**

We sequentially analyzed the amplification of APO-B, D1S80, DxS52, FVW, 33.6, YNZ-2 and H-ras primers using variable number of tandem repeats (VNTR) on 17 pairs and fluorescent *in situ* hybridization (FISH) with the XY probe and SRY primer on 13 sex-unmatched pairs.

**RESULTS:**

The informativeness of the primers using VNTR was 60% for APO-B, 75% D1S80, 36% DxS52, 14% FVW, 40% YNZ-22 and 16% H-ras. The SRY primer was informative in female receptors with male donors. The XY-FISH method was informative in 100% of the sex-unmatched pairs.

**CONCLUSION:**

These methods were sensitive and informative. In VNTR, the association of APO-B with D1S80 showed 88% informativeness. The quantitative FISH method was more sensitive, but had the disadvantage of only being used for sex-unmatched pairs.

## RESUMO

**CONTEXTO E OBJETIVO:**

O transplante de células hematopoiéticas não-mieloablativo é realizado em pacientes com doenças onco-hematológicas que não suportam condicionamentos ablativos devido à elevada idade ou ao acometimento por comorbidades. Esta abordagem não elimina completamente as células do hospedeiro, resultando, inicialmente, em quimerismo misto. A persistência do quimerismo misto na evolução de longo prazo resulta na rejeição ao enxerto e recaída. O acometimento pela doença do enxerto contra hospedeiro é concomitante ao quimerismo completo e ao efeito enxerto *versus* tumor. O objetivo deste estudo foi avaliar a prevalência do quimerismo em doenças onco-hematológicas tratadas com o transplante não-mieloablativo de células hematopoiéticas.

**TIPO DE ESTUDO E LOCAL:**

Estudo clínico observacional do estado de quimerismo após transplante antígenos leucocitários humanos-idêntico não-mieloabaltivo realizado na Disciplina de Hematologia e Hemoterapia da Universidade Federal de São Paulo.

**MÉTODOS:**

Analisamos sequencialmente a amplificação dos primers APO-B, D1S80, DxS52, FVW, 33,6, YNZ-22, H-ras pelo VNTR (variable number of tandem repeats) em 17 pares e FISH (fluorescent *in situ* hybridization) pela sonda XY e do primer SRY em 13 pares de não relacionados a sexo.

**RESULTADO:**

A informatividade dos *primers* pelo VNTR foi de 60% para APO-B; 75% D1S80; 36% DxS52; 14% FVW; 40% YNZ-22 e 16% H-ras. O *primer* SRY foi informativo em receptores femininos com doadores masculinos. O método XY-FISH foi informativo em 100% dos pares de não relacionado a sexo.

**CONCLUSÃO:**

Estes métodos foram sensíveis e informativos. No VNTR, a associação do APO-B com D1S80 mostrou 88% de informatividade. O FISH, método quantitativo, foi mais sensível, porém com desvantagem de ser usado somente nos pares não relacionados a sexo.

## INTRODUCTION

Non-myeloablative hematopoietic stem cell transplantation (NMA-HSCT) is indicated for patients who cannot tolerate conventional ablative regimens because of older age or the presence of comorbidities. Non-myeloablative regimens do not completely eliminate the cells from the host. Initially, both donor and recipient cells are present, a condition known as mixed chimerism. This state of mixed chimerism is subsequently converted into complete chimerism through infusions of the donor’s lymphocytes or manipulation of immunosuppression during the post-transplant period. The persistence of mixed chimerism, regardless of whether ablative or non-myeloablative conditioning is performed, results in relapses.^1^^,^^2^^,^^3^

Host involvement through graft-versus-host disease generally occurs concurrently with complete chimerism. Successful clinical manipulation of graft-versus-host disease is desirable because it is usually associated with eradication of the underlying disease through the graft-versus-tumor effect. Full chimera means complete chimerism of immune and hematopoietic cells, consisting of 100% donor cells, and is the main factor for achieving definitive cure.

After transplantation, chimerism analysis provides important clinical information, such as in relation to total or partial reconstitution of the immune system and hematopoiesis in grafted cells. Evaluations on the polymerase chain reaction (PCR), markers for polymorphism loci of variable number tandem repeats (VNTR) and short tandem repeats (STR) inform the degree of chimerism in sex-matched and sex-unmatched transplants. These techniques allow assessment of both pairs, or differentiation between donor and host deoxyribonucleic acid (DNA). The fundamental principle of the VNTR and STR techniques is that they distinguish DNA patterns between the donor and host by means of a panel of specific primers prior to transplantation. Once such a panel has been established, post-transplant monitoring of chimerism becomes applicable. The intensity of chimerism that is found depends on identifying and measuring genetic markers that vary between the donor and host.^3^^,^^4^

The conventional cytogenetic analysis that is classically used to assess chimerism following sex-unmatched transplantation is inappropriate because only small numbers of cells undergoing mitosis are available after transplantation. This difficulty is explained by the hypocellularity of the bone marrow, which can persist for months after the infusion, as well as by difficulties in inducing mitosis using the usual mitogens.^1^^,^^5^^,^^6^

Mixed chimerism is defined as the presence of donor cells in proportions between 2.5 and 97%, within the cellular context of host hematopoiesis. XY fluorescent *in situ* hybridization (FISH) is used on sex-unmatched pairs as a simple quantitative method for investigating this, and is applicable even when there are low numbers of cells in marrow samples.^7^ The XY probe allows two different colors to be viewed.^8^ Its resolution is high, but it can only be applied in cases of sex-unmatched transplants.^6^ The SRY marker can also be used to quantify the presence of the Y chromosome in sex-unmatched pairs.^9^^,^^10^^,^^11^

NMA-HSCT can be performed with the patient either in the ward or in the outpatient clinic.^12^^,^^13^^,^^14^ The classical indications for non-myeloablative regimens are indolent disease such as chronic myeloid leukemia, low-grade non-Hodgkin’s lymphomas, chronic lymphocytic leukemia and mantle cell lymphoma.^14^^,^^15^^,^^16^^,^^17^^,^^18^^,^^19^ These diseases are intensely sensitive to the graft-versus-host effect. The diseases that present intermediate sensitivity to this immunological effect are acute myeloid leukemia, intermediate and high-grade non-Hodgkin’s lymphomas, multiple myeloma and Hodgkin’s lymphoma.^12^^,^^14^^,^^15^^,^^16^^,^^17^^,^^18^^,^^19^

## OBJECTIVE

The aim of this study was to evaluate the prevalence of chimerism in 21 patients with onco-hematological diseases who underwent NMA-HSCT and the resolution of different techniques.

## MATERIALS AND METHODS

### Patient sample

This was a case series comprising 21 patients (14 males and seven females), with a mean age of 47 years and an average of two years of disease evolution prior to the transplantation. Three of the patients had undergone autologous stem cell transplantation previously. Two of the patients underwent transplantation presenting with positive minimal residual disease, seven with progressive disease and two with refractory disease.

All of the patients were included consecutively in this study, without selection, between February 2003 and January 2006. Twelve patients were from Hospital Santa Marcelina and nine from Hospital São Paulo. Two donor/recipient sex-unmatched patients were subsequently included for XY-FISH analyses in 2006. All of the patients underwent matched HLA (human leukocyte antigen)-identical NMA-HSCT. The non-myeloablative conditioning regimen protocols included 14 of the 21 patients, who used fludarabine in their regimens.^13^^,^^15^^,^^16^^,^^17^^,^^18^^,^^19^ Six multiple myeloma patients received melphalan doses lower than 140 mg/m² and one received 120 mg/m² plus 120 mg/m² of cyclophosphamide as their conditioning.^15^ The prophylaxis for graft-versus-host disease consisted of cyclosporine-A plus methotrexate for all settings.^17^^,^^18^ The infectious disease prophylaxis that was used followed the universally known protocols.^15^^,^^16^^,^^17^^,^^18^^,^^19^ This study was previously approved by the Research Ethics Committees of both institutions (Protocol numbers 18/2005 and 1162/03 for Hospital Santa Marcelina and Hospital São Paulo, respectively) and an informed consent statement was signed by all patients and their donors.

Table 1 presents the characteristics of the patient sample: numbers of patients, clinical situation (underlying diseases and patients’ clinical situation before transplantation), recipient and donor genders, comorbidities, age at the time of transplantation and duration of illness until the transplantation was performed.

**Table 1.** Characteristics of patient sample: numbers of patients, underlying disease, age at the time of transplantation, recipient and donor genders, comorbidities and duration of illness until transplantation was performed

Patient	Disease	Age (years)	Sex R/D	Pre-HSCT status	Comorbidities	Evolution (months)
4	MM	28	M/F	PD, MM-IIIA	-	10
8	MM	56	M/F	PD, post-AHSCT, MM-IIIA	-	44
10	MM	53	M/F	MM-IIIA	CRF	18
12	MM	53	F/M	MM-IIIA	OB	12
17	MM	46	M/M	PD, MM-IIIA	-	8
18	MM	48	M/M	2^nd^ PR, MM-IIIB	-	5
09	MM	49	F/M	PD, post- AHSCT, MM-IIIA	-	69
15	MM	42	F/F	PD, MM-IIIA	DVT-LL	11
14	C-CML	39	F/F	C-CML	SKS, CRRF	36
3	MDS	55	M/F	7q-(T-RAEB), MDS	CA-HCV	6
6	AML-M3	33	F/M	MRD, AML-M3	CCF*	18
7	AML-M2	20	M/F	Recurrence post-AHSCT, AML-M2	CCF*	8
13	MDS	63	M/F	5q-RAEB, MDS	DM, HT	30
16	AML	59	M/M	2^nd^ MDS, 1^st^ CR, AML	CCC, HT	13
21	C-CML	53	M/M	C-CML	CCF	24
1	CLL	47	M/F	Binet C, MRD, CLL	-	16
2	FL	44	M/F	Relapsed IVB, FL-GII,	-	36
5	NHL	38	M/F	RD rich in T cell, IVB, DLBCL	AC, HPV	33
11	NHL	58	F/M	DP, post-AHSCT, MM-IIIB, GII, FL	COPD	28
19	CLL, RS	53	M/M	DP, CLL, RS	CA-HBV, DM, HT, OB	96
20	NHL	48	F/F	MF	HT	10

IVB = stage IVB (Ann Arbor), bone marrow or liver disease infiltration; AML-M3 = promyelocytic acute myeloid leukemia; AML-M2 = acute myeloid leukemia with differentiation (FAB-M2); RAEB = refractory anemia with excess of blasts; MM-IIIA or B = multiple myeloma with Durie & Salmon stage III A (without renal failure) or B (with renal failure); FL-GII = follicular lymphoma grade II; CRF = chronic renal failure; DVT-LL = deep vein thrombosis of lower limbs; Sex R/D = sex of recipient/donor; M = Male; F = Female; PD = progressive disease; RD = refractory disease; AHSCT = autologous hematopoietic stem cell transplantation; OB = obesity; MRD = minimal residual disease; CR = complete remission; PR = partial remission; CCF = congestive cardiac failure, CCF* = congestive cardiac failure secondary to use of anthracyclines; CA-HBV = chronic active hepatitis B virus, CA-HCV = chronic active hepatitis C virus; HT = hypertension; CCC = chronic calculous cholecystitis; DM = diabetes mellitus; SKS = severe kyphoscoliosis; CRRF = chronic restrictive respiratory failure; COPD = chronic obstructive pulmonary disease; FL = follicular lymphoma; DLBCL = diffuse large B cell lymphoma; MF = mycosis fungoides; MDS = myelodysplastic syndrome; AC = alcoholic cirrhosis; RS = Richter syndrome; HPV = herpes papillomavirus, C-CML = chronic phase of chronic myelogenous leukemia; NHL = non-Hodgkin’s lymphoma; CLL = chronic lymphocytic leukemia.

### Chimerism prevalence evaluation

This was a cross-sectional prevalence study on chimerism. We used the VNTR technique on 17 pairs and FISH with XY probe on sex-unmatched pairs. The chimera evaluations were done sequentially prior to transplantation in order to define the different primer pairs between the donor and recipient. DNA amplification was done on the patients on days 30, 60, 120, 180 and 360, and subsequently, much later after transplantation (+1200 days), on two patients. The results from the VNTR technique were correlated with those from FISH.

We used the VNTR technique to analyze the DNA obtained from the total peripheral blood leukocytes of the donor and recipients. Markers were selected based on their characteristics, degree of resolution or presence of polymorphism in multiple alleles, with homogeneous distribution frequency in populations previously studied ^3^^,^^6^^,^^18^^,^^19^^,^^20^^,^^21^^,^^22^^,^^23^^,^^24^^,^^25^^,^^26^^,^^27^^,^^28^^,^^29^^,^^30^^,^^31^ and consequently a high degree of heterozygosity (Table 2). The SRY marker was chosen for examining transplantations from sex-unmatched donors, in order to determine whether the Y chromosome was present or absent.^10^^,^^11^ XY-FISH was performed on total bone marrow cells in the first and final evaluation of chimerism after transplantation from sex-unmatched donors.^8^

DNA from recipients and donors was extracted using the Perfect gDNA Blood Mini-isolation kit (Eppendorf, Germany).^22^ It was amplified using the polymerase chain reaction (PCR) technique, by means of a thermocycler (Perkin Elmer 9700), for eight pairs of specific primers for each sequence. The characteristics of the chromosomal locations, base pair sequences and sizes of the amplified fragments are shown in Table 3 and the amplification conditions are shown in Table 4.

**Table 2.** Markers according to amplification and resolution

Marker	DNA amplification	Resolution
APO-B	16/17 (94%)	10/16 (62.5%)
DS180	15/17 (88%)	10/15 (75%)
DxS52	11/17 (64%)	4/11 (36%)
FVW	14/17 (82%)	2/14 (14%)
33.6	10/17 (58%)	4/10 (40%)
YNZ-22	15/17 (88%)	7/15 (47.6%)
H-ras	6/17 (35%)	1/6 (16.6%)
All markers	17/17 (100%)	16/17 (94%)

**Table 3.** Primer sequences used in variable number of tandem repeats (VNTR) technique

Primer	Chromosome location	Primer sequence	Product (base pairs)	Reference
APO-B	2p23 - p24	5’ ATG GAA ACG GAG AAA TTA TG 3’	500 to 900	21
		5’ CCT TCT CAC TTG GCA AAT AC 3’		
D1S80	1p35 - p36	5’ GAA ACT GGC CTC AAC ACT GCC CGC C 3’	250 to 650	25
		5’ GTC TTC TTG GAG GCA CGT GCC CCT T 3’		
DxS52	Xq26 - q28	5’ CGA AGA GTG AAG TGC ACA GG 3’	650 to 3000	23
		5’ CAC AGT CTT TAT TCT TCA GCG 3’		
FVW	12p13-3	5’ AGC TAT ATA TCT ATT TAT CAT 3’	300 to 700	26
		5’ ACA TAC ATA CAT AGA TAT AGG 3’		
33.6	1q	5’ TGT GAG TAG AGG AGA CCT CAC 3’ 5’AAA GAC CAC AGA GTG AGG AGC 3’	500 to 1000	27
H-ras	11p15.5	5’ TTG GGG GAG AGC TAG CAG GG 3’ 5’ CCT CCT GCA CAG GGT CAC CT 3’	1000 to 2600	27
YNZ-22	17p13.3	5’ GGT CGA AGA GTG AAG TGC ACA G 3’ 5’ GCC CCA TGT ATC TTG TGC AGT G 3’	200 to 2000	27
SRY	Y	5’ TCG CGA TTA AGT CAA ATT CGC 3’ 5’ CCC CCT AGT ACC CTG ACA ATG TAT T 3’	136	24

**Table 4.** Conditions for primer amplification

Marker	Denaturing	Girdling/extension (temperature, time, number of cycles)		Reference
APO-B	94 °C, 1 min	58 °C (6 min)	x 26	28
D1S80	94 °C, 1 min	65 °C (1 min); 70 °C (5 min)	x 28	29
DxS52	94 °C, 20 sec	55 °C (30 sec); 74 °C (20 sec); final 74 °C (5 min)	x 24	23
FVW	94 °C, 20 sec	48 °C (20 sec); 72 °C (20 sec)	x 30	26
33.6	95 °C, 1 min	60 °C (45 sec); 72 °C (1 min); 72 °C (10 min)	x 35	22
H-ras	94 °C, 5 min	95 °C (1 min); 64°C (2 min); 72 °C (6 min) 64 °C (2 min); 72 °C (10 min)	x 20 x 01	30
YNZ-22	95 °C, 1 min	60 °C (45 sec); 72 °C (1 min); 72 °C (10 min)	x 30	22
SRY	95 °C, 10 min	95 °C (15 sec); 60 °C (1 min); 72 °C (1 min); final 72 °C (7 min)	x 50	24

### XY-FISH

The XY-FISH method was carried out using fresh material. We collected 3 to 5 ml of bone marrow samples into syringes containing sodium heparin. We then removed the leukocyte layer, which had been fixed in Carnoy’s solution, and subsequently prepared microscope slides. These were denatured and hybridized in accordance with the protocol from the probe manufacturer (Vysis, Inc, United States). The slides were evaluated under an epifluorescence microscope with diamidinophenylindole (DAPI), fluorescein isothiocyanate (FITC) and rhodamine filters. The images were captured using the MacProbe 4.4 computer software (PowerGene System, Applied Imaging Corporation, United States) and scanned. The red (rhodamine) signal corresponded to the region of the Y chromosome and green (fluorescein) signal to the region of the X chromosome. Cells with two green signals were interpreted as female (XX) and cells with one green and one red signal, as male (XY).^8^

## RESULTS

The VNTR technique was evaluated for 17 of the 21 patients. Four patients (# 1, 2, 9 and 13) were only analyzed using the FISH method with XY probe. Among these four, cases 1 and 2 were analyzed 1200 days after the transplant. Case 9 was analyzed on days 60 and 360, and case 13 only on day 360. Cases 1 and 2 did not form part of the first period of this study and, in cases 9 and 13, no donor/recipient DNA analysis was obtained prior to transplantation, but in all these cases, the probe evaluated using XY-FISH documented complete chimerism. Even though these cases were not evaluated using the VNTR technique prior to transplantation, the complete chimerism found using the XY probe was confirmed by the SRY marker. The other sequence primers were found to be the following after transplantation: APO-B (cases 1, 2, 9 and 13), D1S80 (1, 2, 9 and 13), FWF (1 and 2), 33.6 (1 and 2), YNZ-22 (1, 2, 9 and 13), DxS52 (9) and H-ras (9).

A panel of markers consisting of APO-B, D1S80, YNZ-22, 33.6, DxS52, FVW, H-ras and SRY was used for the other 17 patients and the results obtained are described below.

Case 3 was an extension of DNA fragments that were identical for recipient and donor, to the APO-B, D1S80, DxS52, FWF and 33.6 markers. The donor and recipient bands for the YNZ-22 and H-ras markers failed to be obtained. A review using XY-FISH on day 30, showed mixed chimerism with XX cells accounting for 85%. The donor for this particular patient was his mother.

Evaluation of the product from APO-B showed results in 16 cases (94%), with information only on 10/16 patients (62.5%). Cases 5, 6, 8, 10, 12, 14, 16, 17 and 22 showed different bands for these markers. It should be noted that patients 5, 16 and 21 only showed the APO-B marker through the VNTR technique (Table 2).

With the D1S80 marker, amplification occurred in relation to 15 patients (88%); but information was found relating to 10/15 (75%). For 5/17 (29%), informative bands were present using the APO-B and D1S80 primers (cases 6, 8, 12, 14 and 17). On the other hand, regarding the panel composed only of the D1S80 and APO-B primers, resolution of chimerism occurred in relation to 15/17 pairs (88%). Only cases 3 and 20 showed no information relating to either of these two markers.

With the YNZ-22 marker, amplification was observed in relation to 15/17 (88%), but information was seen relating to 7/15 (47.6%). Patients 4, 6, 8, 10, 11, 12 and 18 showed information with this marker. Case 18 was also evaluated using the STR primer (not shown), presenting complete chimerism through both methods at the same time.

Using the DxS52 marker, amplification showed a DNA product for 11/17 patients (64%), but information relating to this marker was seen in only four pairs (36%): patients 4, 7, 11 and 19. With the FVW marker, amplification was seen in relation to 14/17 (82%), but information from this marker was documented in only two patients (14%): cases 4 and 7. Using the 33.6 marker, amplification was seen in relation to 10/17 (58%) and there was pretransplantation information for 4/10 (40%). Cases 7, 10, 12 and 20 presented resolution with this marker. It should be noted that case 20 only had the 33.6 primer as a marker.

**Figure 1:**
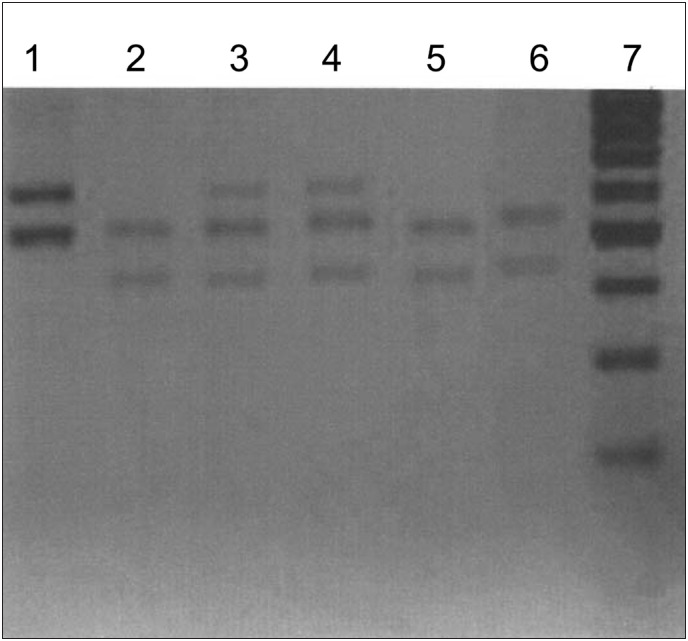
Evaluation using 2% agarose gel electrophoresis marker D1S80 following polymerase chain reaction (PCR) amplification (variable number of tandem repeats, VNTR technique) in case 7. (1) donor product; (2) patient product before transplantation; (3) and (4) donor and patient products after transplantation; (5) and (6) patient productsafter transplantation and after molecular relapse; (7) 100-base pair marker.

Case 7 showed mixed chimerism documented using XY-FISH, with only 34% of the cells coming from the donor. This finding was confirmed using the D1S80, DxS52 and 33.6 primers. With the FVW marker, there was no grafting, and this was a different result. The interpretation was that this was mixed chimerism due to the other primers. On day 90, this patient presented molecular relapse, which was confirmed by information from all of the primers investigated. It should be noted that no acute or chronic graft-versus-host disease involvement was seen in this patient. Hematological relapse occurred 120 days after transplantation (Figure 1).

XY-FISH analysis on day 30 showed that 99% of the cells were from the donor in cases 4 and 10 and 97% in cases 5 and 12. Analysis using the VNTR technique concomitantly showed that case 4 presented complete chimerism with D1S80, DxS52, FVW and YNZ-22; case 5 with APO-B; case 10 with APO-B, 33.6 and YNZ-22; and case 12 with APO-B, D1S80, 33.6, YNZ-22 and SRY.

Evaluation using the SRY primer on female recipients with male donors enabled analysis on four cases and documented complete chimerism in three of them (cases 6, 11 and 12). On the other hand, in case 9, this marker and other markers were not evaluated on day 30 but, as mentioned earlier, XY-FISH documented complete chimerism on day 60.

With regard to male recipients with female donors, the SRY marker did not give information for analyzing chimerism, because all these recipients had the Y chromosome, regardless of the time at which they were examined and regardless of the outcome of complete chimerism diagnosed using XY-FISH.

Thirteen patients were analyzed using XY-FISH. Of these, four were female recipients (# 6, 9, 11 and 12) and seven were male (# 1, 2, 3, 5, 6, 7, 8, 10 and 13). This was the most sensitive method, and it allowed evaluation of chimerism in patients 1, 3, 2, 9 and 13, who had not been investigated regarding DNA prior to transplantation. From the SRY marker, it was seen that the female recipients kept the male Y chromosome, thus leading to an interpretation of complete chimerism, in combination with the XY-FISH probe results.

## DISCUSSION

The resolution found using APO-B was 10/16 (62.5%). It was noteworthy in relation to this set of primers that cases 5, 16 and 21 only had the APO-B primer as a marker for chimerism. Stuppia et al.^25^ documented resolution in 25% of Caucasian pairs using D1S80. According to Martinelli et al.,^20^ D1S80 was the main marker in their cases with resolution of 6/6 cases. Muniz et al.^31^ found 66% resolution. In our series, amplification occurred in 88% of the cases (15 pairs) and resolution in 10/15 (75%). We emphasize the fact that 5/17 patients (29%) presented resolution using the APO-B and D1S80 primers, but when we summed the separate results, combining them for both primers, there was information on 15 of the 17 pairs (88%). Thus, combination of these two primers had great applicability in our series.

DxS52 was investigated by Stuppia et al.,^25^ and they obtained resolution in 4/6 cases (66.6%). There was successful amplification of the DNA fragment from 11/17 pairs (64%), at different times after transplantation, but the degree of resolution among our patients was 4/11 (36%), i.e. lower than that described by Stuppia et al.^25^

FVW was analyzed in relation to 35 pairs by Muniz et al.^31^ and resolution was detected in 15 (43%). In our series, despite the success of amplification in relation to 14/17 patients (82%), there was resolution in only 2/14 (14%). This was also seen with H-ras, which gave information only in the case of one patient (case 17), and there was difficulty in amplification in 11/17 cases (65%). Our data for H-ras therefore differ from those of Hassan et al.,^30^ who observed 36% resolution in cases of aplastic anemia, and were closer to the data of van Leeuwen et al.,^32^ who found resolution relating to only 11% of 37 pairs.

We succeeded in amplifying YNZ-22 in 15/17 pairs (88.8%), and the resolution was 7/15 (47%). Hassan et al.^30^ found that the resolution of this primer was 50%, among individuals with aplastic anemia. The 33.6 primer gave information in relation to 40% (4/10), and this percentage was lower that described by Hassan et al.^30^

The resolution found for this panel of primers was as follows: D1S80, 10/15 patients (75%); APO-B, 10/16 (62.5%); YNZ-22, 7/15 (47%); 33.6, 4/10 (40%); DxS52, 4/11 (36%); FVW, 2/14 (14%); and H-ras, 1/6 (16.6%). YNZ-22 and FVW were easily amplified; YNZ-22 showed intermediate resolution, but FVW showed the lowest heterozygosity. We stress the importance of using larger numbers of primers in such panels, in order to achieve relevant results. Case 3 provides an example: this showed mixed chimerism through XY-FISH but the VNTR technique failed to show chimerism, since successful amplification of the APO-B, D1S80, DxS52, FVW and 33.6 primers showed homozygous bands. The importance of this panel was shown by the fact that patients 5, 15, 16, 20 and 21 presented only one primer marker. APO-B was the marker in cases 5, 16 and 21; D1S80 in case 15; and 33.6 in case 20.

The informativeness of each individual locus in VNTR/STR varies between different individuals and, therefore, the polymerase chain reaction products are of different lengths depending on the tandem repeats. Currently, DNA-based technologies are the methods of choice for chimerism analysis, mainly because of their sensitivity for detecting the presence of minor proportions of recipient clone cells after transplantation. Ariffin et al.^4^ evaluated individuals of Malay and Chinese ethnicity using two commercially-available forensic kits that are also widely used for multiplex and monoplex STR. In their cases, amplification and genotyping using this method revealed six out of 15 STR loci, which enabled resolution of 31 out of 33 cases (94%): TH01 (73%), VWA (73%), FIA0I (52%), CSFIPO (61%), FESFPS (39%) and TPOX (45%).^4^ A combination of STR and VNTR markers tested by Talwar et al.^3^ in relation to hematopoietic stem cell transplantation cases and normal subjects in northern India demonstrated that the APOB’HVR, FES, VWA, D3S1358 and D16S310 loci were the most polymorphic of the 15 loci analyzed, with mean heterozygosity of 0.756 ± 0.17. A panel composed of these seven primers was able to differentiate 98.7% of the donor-recipient pairs out of the 77 settings tested. We emphasize that APO-B and D1S80 made resolution possible for 88% of our patients, but a larger population of both normal individuals and hematopoietic stem cell transplantation cases needs to be evaluated in order to reach a final conclusion.

The FISH XY probe was used in the cases of 13 patients, with the first sample on day 30 and the second concomitantly with the last collection for the VNTR technique. For four patients (1, 2, 9 and 13), no DNA analysis was performed prior to transplantation and although the VNTR technique showed homozygous bands, the final chimerism analysis was certified by means of XY-FISH.

The sensitivity of the XY-FISH method ranges from 0.7 to 5%.^6^ Two out of four patients had FISH values of 99 and 99.5% on day 30 (cases 4 and 10, respectively). In case 4, the D1S80, DxS52, FVW and YNZ-22 primers showed qualitative results with complete chimerism and in case 10, the APO-B, 33.6 and YNZ-22 primers presented equal results. Both of these patients displayed complete chimerism in subsequent evaluations, on days 60, 90, 120, 180 and 360 in case 10 and on all these occasions plus day 1200 in case 4. For patients 5 and 12, XY-FISH showed that the proportions of donor cells were 97 and 97.5%, respectively, in the evaluation on day 30. Patient 5 only had the APO-B primer as a marker, while patient 12 had APO-B, D1S80, 33.6 and YNZ-22. On day 30, both of these patients presented complete chimerism. Further evaluations, which continued until day 180 for patient 12 and until day 120 for patient 5, documented complete chimerism according to the VNTR technique. For these two patients, XY-FISH at the last evaluation was also compatible with complete chimerism. In all the other evaluations using the XY probe, totaling 19 simultaneous analyses, the degree of chimerism was coincident with the results for primer markers using the VNTR technique, which included the interpretation of day 30 for cases 4 and 10. From these results, it can be concluded that the XY probe, as assessed using FISH, which is feasible only for sex-unmatched pairs, is a more sensitive method than the VNTR technique, especially for early assessments following transplantation. Thus, for the NMA-HSCT technique, XY-FISH is important because of its outstanding capability for determining whether or not to perform donor lymphocyte infusion (DLI) in order to obtaining complete chimerism.

It should be noted that the analysis of hematopoietic chimerism using the XY probe does not depend on data collection prior to transplantation, as does the VNTR interpretation. The interpretation of XY probe data is quantitative and more decisive than is the qualitative or semiquantitative assessment of VNTR data. Today, single nucleotide polymorphism, STR and Y-chromosome-specific sequences analyzed by polymerase chain reactions (PCR) are the best methods for carrying out a quantitative evaluation of chimerism. Koldehoff et al.^11^ reported that single nucleotide polymorphism using 10 different gene loci discriminated patient cells from donor cells in 125 out of 135 pairs (93%), while STR performed with 11 different genes resulted in accurate donor-host discrimination in all pairs. The results from the two methods were 74% concordant, but the single nucleotide technique detected mixed chimera earlier than STR did, thus enabling earlier interventions. Using a multivariate Cox model, this method was the only significant factor predicting relapse.

Studies on chimerism are of fundamental importance for the final diagnosis to explain why rejection occurs and for clarifying the etiology of hypoplasia following transplantation and any recurrences of the underlying disease. In this study, we evaluated the presence of SRY using normal controls consisting of male and female individuals who had not undergone transplantation. We observed four cases of female recipients with male donors, showing complete chimerism according to XY-FISH. This result was identical to that obtained through analysis of SRY in the DNA of blood cells. Meanwhile, seven male patients with female donors presented complete chimerism according to XY-FISH, but they were positive for SRY, from evaluation of the DNA of their blood cells. The control that we used in each amplification reaction was DNA from normal males and females, but even if different samples were evaluated at different times, the results from these patients were always convergent using the SRY technique and divergent using the FISH technique. Koldehoff et al.^11^ analyzed the status of male recipients with female donors in 580 samples from 134 patients, using a quantitative real-time polymerase chain reaction (RT-PCR) on Y-chromosome specific sequences. They compared the results with interphasic XY-FISH and demonstrated the presence of mixed chimerism without signs of relapse in 35% of the samples. Otherwise, the quantities of Y-DNA detected were low, compared with the amounts detected in 104 samples from relapsed leukemia patients at the time of analysis. Importantly, the amount of host DNA increased sequentially in relapsed patients, thereby proving that mixed chimerism becomes enhanced and possibly predicts relapse (which occurred after approximately 143 days in this study). The results were concordant with XY-FISH findings in 73% of the patients. However, XY-FISH detected relapses only in 12 settings and Y-chromosome polymerase chain reactions detected 36, by means of sequential evaluation of mixed chimerism. Based on our results, we ruled out the use of quantitative methods for evaluating chimerism in male recipients using the SRY primer, and the results must be interpreted with caution.

## CONCLUSION

1. The VNTR technique enabled evaluation of chimerism in 17 out of 21 patients, and the primers used presented the following resolution: APO-B (62.5%), D1S80 (75%), DxS52 (36%), FWF (14%), 33.6 (40%), YNZ-22 (47%) and H-ras (16.6%).
2. The XY-FISH technique, which was used for 13 cases in which the donor and recipient were of different genders, showed quantitative resolution of chimerism in 100% of cases.
3. In two patients, mixed chimerism was identified. In these pairs, there were discrepancies in the assessment of chimerism on day 30 between the XY-FISH and VNTR techniques. XY-FISH identified 95% and 97% mixed chimerism and the VNTR technique showed complete chimerism at this stage of the procedure. In other patients and at other times, the two methods were coincident.
4. The SRY primer proved to be capable of resolution only in cases of female recipients when their results were coincident with those from XY-FISH.
5. When the APO-B and D1S80 primers were considered in the panel, this enabled resolution of chimerism in 15 out of 17 patients (88%).
